# Retinal Ganglion Cell Loss is Delayed Following Optic Nerve Crush in NLRP3 Knockout Mice

**DOI:** 10.1038/srep20998

**Published:** 2016-02-19

**Authors:** Zhen Puyang, Liang Feng, Hui Chen, Peiji Liang, John B. Troy, Xiaorong Liu

**Affiliations:** 1 School of Biomedical Engineering, Shanghai Jiao Tong University, 800 Dong-chuan Road, Shanghai 200240, China; 2 Department of Biomedical Engineering, Robert R. McCormick School of Engineering and Applied Science, Northwestern University, Evanston, Illinois 60208, USA; 3 Department of Ophthalmology, Feinberg School of Medicine, Northwestern University, Chicago, Illinois 60611, USA; 4 Department of Neurobiology, Weinberg College of Arts and Sciences, Northwestern University, Evanston, Illinois 60208, USA

## Abstract

The NLRP3 inflammasome, a sensor for a variety of pathogen- and host-derived threats, consists of the adaptor ASC (Apoptosis-associated Speck-like protein containing a Caspase Activation and Recruitment Domain (CARD)), pro-caspase-1, and NLRP3 (NOD-Like Receptor family Pyrin domain containing 3). NLRP3-induced neuroinflammation is implicated in the pathogenesis and progression of eye diseases, but it remains unclear whether activation of NLRP3 inflammasome contributes to retinal ganglion cell (RGC) death. Here we examined NLRP3-induced neuroinflammation and RGC survival following partial optic nerve crush (pONC) injury. We showed that NLRP3 was up-regulated in retinal microglial cells following pONC, propagating from the injury site to the optic nerve head and finally the entire retina within one day. Activation of NLRP3-ASC inflammasome led to the up-regulation of caspase-1 and a proinflammatory cytokine, interleukin-1β (IL-1β). In NLRP3 knockout mice, up-regulation of ASC, caspase-1, and IL-1β were all reduced, and, importantly, RGC and axon loss was substantially delayed following pONC injury. The average survival time of RGCs in NLRP3 knockout mice was about one week longer than for control animals. Taken together, our study demonstrated that ablating the *NLRP3* gene significantly reduced neuroinflammation and delayed RGC loss after optic nerve crush injury.

Growing evidence suggests that activation of microglia, the resident immune cells of the central nervous system, initiates the destructive inflammatory process resulting in vision loss in eye diseases (reviewed by^1^,^2^,^3^,^4^). Microglial hyperactivity has been found to be associated with photoreceptor degeneration^5^,^6^, retinal pigment epithelial (RPE) damage in age-related macular degeneration (AMD)^7^,^8^,^9^,^10^, as well as retinal ganglion cell (RGC) loss in experimental glaucoma^11^,^12^,^13^,^14^,^15^,^16^.

The NLRP3 inflammasome in microglia and macrophages is activated in response to diverse stimuli including microbial and other environmental perturbations^17^. Activation of the NLRP3 inflammasome leads to activation of caspase-1 protease, resulting in cleavage of pro-interleukins, a well-characterized inflammatory response^17^. The proinflammatory molecules have been detected in patients with eye diseases^18^,^19^, in rodent models of experimental glaucoma^20^,^21^, and mouse models of AMD^7^,^8^,^9^,^10^. Some studies showed that activation of the NLRP3 inflammasome induced a gradual breakdown of macula RPE in dry/atrophic AMD^9^,^10^. Another study suggested, however, a protective role of NLRP3 inflammasome in wet/neovascular AMD, where abnormal blood vessels grow and hemorrhage between the RPE and the foveal photoreceptors^7^. On the other hand, retinal ischemia induced by acute elevation of intraocular pressure triggered the increase of interleukin-1β (IL-1β) through NLRP3-caspase-1-dependent and -independent pathways^22^, suggesting that the activation of NLRP3 may not be required for retinal damage. In this study, we induced acute retinal ganglion cell (RGC) loss by partial optic nerve crush (pONC) injury, and then tested whether activation of the NLRP3 inflammasome causes RGC loss using NLRP3 knockout mice.

## Results

### NLRP3 is up-regulated in retinal microglia following partial optic nerve crush injury

**Figure 1:**
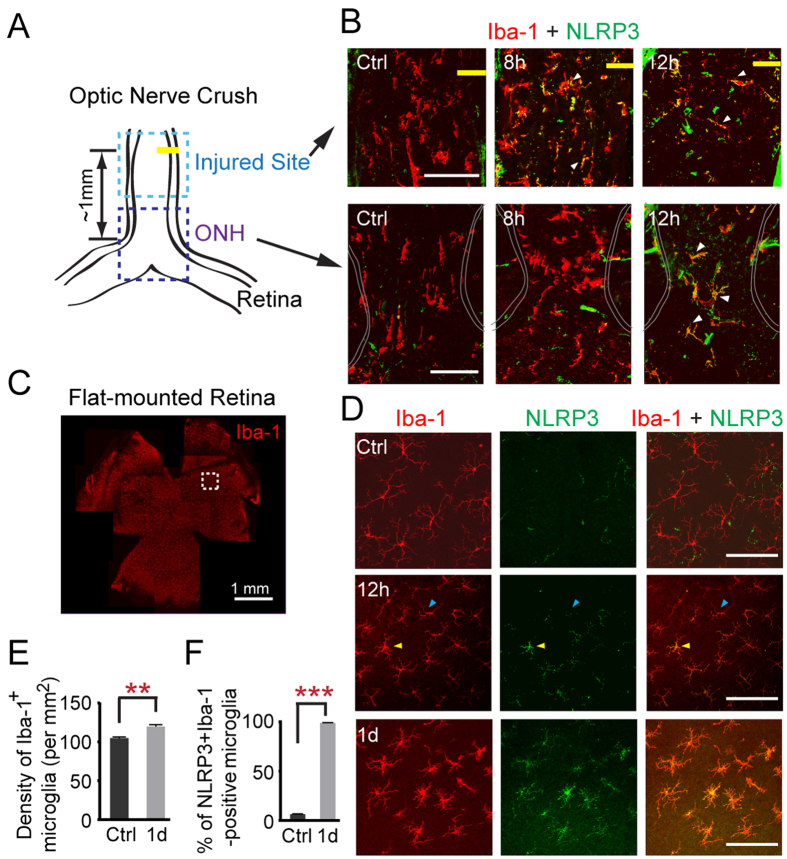
NLRP3 was activated in retinal microglial cells within one day of partial optic nerve crush (pONC). (**A**) Diagram of the optic nerve crush. Yellow bar marks the site of the crush. ONH: optic nerve head. (**B**) NLRP3 was first detected around the site of injury in microglial cells labeled by Iba-1 at 8 hours (h) post pONC, and then propagated to the ONH area at 12-h post pONC. White arrowheads point to the microglial cells double-labeled for NLRP3 and Iba-1. (**C**) Confocal images of a flat-mounted wild-type control (Ctrl) retina immunostained with Iba-1 (red). (**D**) In the control retina, microglial cells in the inner retina were in the inactive state with small nuclei and highly ramified processes; at 12-h post pONC, NLRP3 was detected in some microglial cells (yellow arrowheads), but not in others (blue arrowheads). At 1-day (d) post pONC, NLRP3 was detected in most, if not all, microglial cells of the retina. Some non-specific background staining was seen in blood vessels (top middle), but they were not co-localized with Iba-1+ cells. Scale bars: 100 μm. (**E,F**) The numbers of Iba-1 positive cells (**E**) and cells double-labeled with NLRP3 and Iba-1 (**F**) in the inner retina were significantly increased at 1- d post pONC. N = 4 in each group; **p < 0.01, ***p < 0.001 in Student’s *t*-test.

Acute RGC loss was induced by partial optic nerve crush (pONC) injury (Fig. 1A and Supplementary Fig. S1) and NLRP3 expression examined in retinal microglial cells in mice (Fig. 1B–F). Microglial cells were labeled using an antibody against ionized calcium binding adaptor molecule 1 (Iba-1), a microglia/macrophage-specific calcium-binding protein. In control (ctrl) animals, the expression of NLRP3 was hardly detectable in resting microglia, which had small nuclei and highly ramified processes, of both the optic nerve (Fig. 1B) and retina (Fig. 1C,D). At 8-h post pONC, the co-localization of NLRP3 and Iba-1 was seen near the injured optic nerve, but not around the optic nerve head (ONH, Fig. 1B). At 12-h post pONC, NLRP3 was present at both the injury site and the ONH (Fig. 1B). At the same time, NLRP3 was also detected in some microglial cells in the inner retina (Fig. 1D). At 1-day (d) post pONC, active microglial cells exhibited large nuclei and fewer and shorter processes and NLRP3 was present in most, if not all, microglial cells of the inner retina (Fig. 1D). The number of Iba-1-positive microglial cells increased (n = 4 for each group, p = 0.003 in Student’s *t*-test, Fig. 1E), a sign of inflamed tissue. Moreover, almost all Iba-1-positive microglia expressed NLRP3 at 1-d post injury, compared to controls where there was almost no NLRP3 in Iba-1-positive microglial cells (n = 4 mice, p < 0.001 in Student’s *t*-test; Fig. 1F). Together our results showed that optic nerve crush injury triggered the up-regulation of NLRP3, which propagated from the site of injury to the ONH, and then to the entire retina within one day.

### Activation of NLRP3 inflammasome is necessary for the crush-induced increase of caspase-1 and IL-1β

**Figure 2:**
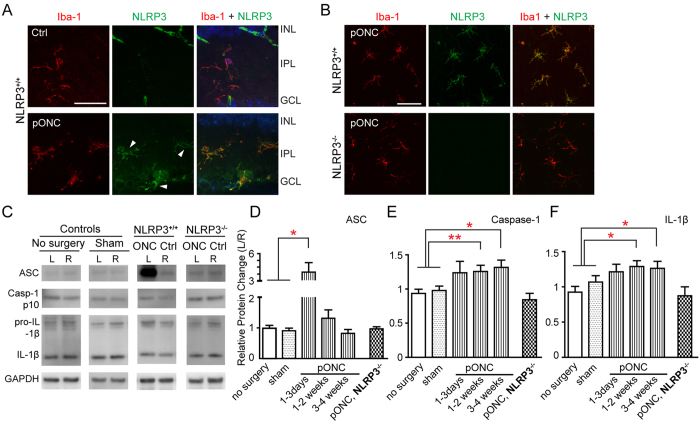
Partial optic nerve crush triggered the up-regulation of ASC, caspase-1, and IL-1β in the mouse retina. (**A**) Confocal images of retinal sections showed the aggregated NLRP3 (puncta staining, arrowheads) in the cytosol of microglial cells following pONC. Nuclear counter-stains by DAPI in merged images were pseudo-colored in dark blue to show the inner nuclear layer (INL) and ganglion cell layer (GCL). IPL: inner plexiform layer. Scale bar: 50 μm. (**B**) The activation of NLRP3 inflammasome was absent in the NLRP3^−/−^ mice at 1-w post pONC. Scale bar: 100 μm. (**C**) Representative Western blots of ASC (~21 Kda), cleaved caspase-1 (~10 kDa), pro-IL-1β (~31 kDa) and IL-1β (~17 kDa), and GAPDH as internal loading controls. The control group included mice without any operation and mice that received sham surgery. For experimental groups, left eyes (L) received the optic nerve crush, and right eyes (R) of the same animals were used as controls. (**D**–**F**) Quantification of the changes of protein levels of ASC (**D**), caspase-1 (**E**), and IL-1β (**F**). The relative protein level was expressed as the ratio of the left and right eyes (L/R ratio). The increase of ASC, caspase-1, and IL-1β was absent in the NLRP3^−/−^ mice post pONC (labeled as pONC, NLRP3^−/−^). N = 5–12 in each group. *p < 0.05, **p < 0.01 in Student’s *t*-test.

One day post pONC, the aggregated puncta staining of NLRP3 in the cytosol of microglial cells indicated the assembly of a large NLRP3 inflammasome complex (Fig. 2A). In NLRP3 knockout mice, no puncta staining of NLRP3 was found in the Iba-1-positive microglial cells following pONC (Fig. 2B). The assembly of the NLRP3 inflammasome leads to the cleavage of pro-caspase-1, which in turn results in production of pro-inflammatory cytokines, such as IL-1β. We examined the protein levels of ASC, capase-1, and IL-1β in mouse retinas post pONC (Fig. 2C–F). Optic nerve crush surgery was performed only on the left eyes, leaving the right eyes (non-operated) to serve as controls. We used the L/R ratio to quantify changes of protein level. When no surgery was performed on either eye, the L/R ratios were close to 1 (ASC: 0.98 ± 0.10, n = 9 mice; caspase-1: 0.94 ± 0.06, n = 12; IL-1β: 0.92 ± 0.08, n = 8; Fig. 2D–F). Similarly, when sham surgery was performed on the left eyes, the L/R ratios were also close to 1 (ASC: 0.91 ± 0.09, n = 10; caspase-1: 0.98 ± 0.07, n = 10; IL-1β: 1.07 ± 0.09, n = 5; Fig. 2D–F). In other words, surgery without optic nerve crush did not induce significant changes in protein level.

We found that ASC was significantly up-regulated following pONC (Fig. 2D), indicating the activation of the NLRP3-ASC inflammasome. The protein level of ASC was more than tripled within three days post pONC (3.92 ± 1.08, n = 6, P < 0.05 in Student’s *t*-test, Fig. 2D)., The level of cleaved caspase-1 was increased at two weeks post pONC (L/R ratio: 1.26 ± 0.09, n = 9 mice, P = 0.01; Fig. 2E) and remained high through the end of four weeks following pONC (1.32 ± 0.11, n = 6, P = 0.02; Fig. 2E). Similarly, the protein level of IL-1β was increased at 2-weeks (1.29 ± 0.08, n = 9, p = 0.009), and 4-weeks post pONC injury (1.26 ± 0.10, n = 9, p = 0.03 in Student’s *t*-test; Fig. 2F).

We next examined whether the protein levels of ASC, caspase-1, and IL-1β would be less when the *NLRP3* gene was knocked out (Fig. 2D–F). Indeed, no increase of ASC, capase-1, or IL-1β was found following the crush injury in the NLRP3^−/−^ mice (ASC: 0.97 ± 0.07, n = 6 mice; caspase-1: 0.84 ± 0.09, n = 7; IL-1β: 0.87 ± 0.13, n = 8; Fig. 2D–F). Our results demonstrate therefore that activation of NLRP3 inflammasome was needed for the increases observed of caspase-1 and IL-1β following the pONC injury.

### Knocking out the *NLRP3* gene delays RGC loss post pONC injury

**Figure 3:**
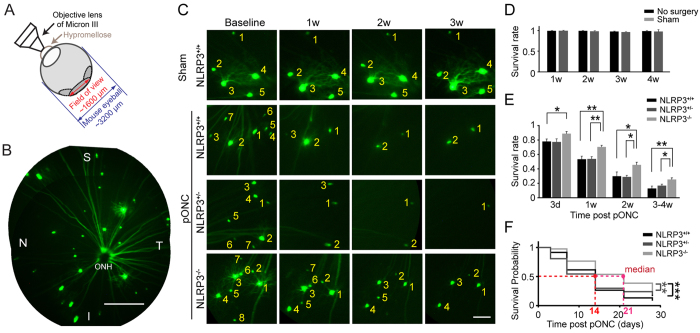
Knocking out the *NLRP3* gene significantly delayed RGC loss following pONC. (**A**) Diagram of *in vivo* imaging with a Micron III scope. (**B**) Multiple images from one Thy-1-YFP transgenic mouse retina were assembled in Photoshop. On average, 14–25 RGCs were seen from one retina. Scale bar: 400 μm. S: superior; I: inferior; N: nasal; T: temporal. (**C**) *In vivo* imaging of the same retinas were taken of NLRP3^+/+^, NLRP3^+/−^, and NLRP3^−/−^ mice at different time points following pONC. RGC somas in serial images of the same area were numbered. Scale bar: 100 μm. (**D**) The number of RGC somas in control animals was not changed over time. (**E**) More RGCs survived in NLRP3^−/−^ mice than in controls (NLRP3^+/−^ and NLRP3^+/+^). *p < 0.05, **p < 0.01 in one-way ANOVA with post LSD test. (**F**) A Kalpan-Meier estimator was applied to determine the probability of RGC survival. Median day of survival was labeled: NLRP3^+/−^ and NLRP3^+/+^: 14 days (red); and NLRP3^−/−^: 21 days (magenta). **p < 0.01, ***p < 0.001 in log-rank test.

To determine whether activation of the NLRP3 inflammasome leads to RGC loss, we tracked RGC survival using the Micron III fundus scope (Fig. 3A–C). As shown in Fig. 3B, RGC somas labeled by the Thy-1-YFP transgene appeared as bright spots in the retina and their axons as bright lines radiating from the ONH. The numbers of RGC somas were counted before surgery, and at different time points post pONC (Fig. 3C). We confirmed that the fluorescent signal was stable over time, and unchanged by the surgery itself (n = 7–15 mice in each point, Fig. 3D). In the NLRP3^+/+^ control mice, only 78 ± 3% of RGCs survived at 3-d post pONC (n = 9 mice, total of 158 cells, Fig. 3E) and RGC loss increased with time: 53 ± 4% of RGCs survived at 1-w and 30 ± 6% at 2-w (Fig. 3E). At 4-w post crush, only 13 ± 3% of cells survived (Fig. 3E). NLRP3^+/−^ mice showed a similar trend (n = 5 mice, total of 86 cells, Fig. 3E). By contrast, more RGCs survived in the NLRP3^−/−^ mice at 3-d post pONC (89 ± 3%, n = 9 mice, total of 181 cells) compared to NLRP3^+/+^ mice (p = 0.03, One-way ANOVA with post LSD test; Fig. 3E). At 1-w post pONC, around 20% more RGCs remained in the NLRP3^−/−^ mice (70 ± 2%) than the other two groups (p = 0.002 compared to NLRP3^+/+^ and p = 0.007 compared to NLRP3^+/−^; Fig. 3E). By the end of 4-w post crush, only 25 ± 2% of RGCs survived in NLRP3^−/−^ mice, but this was much better than for the NLRP3^+/+^ (p = 0.004) and NLRP3^+/−^ mice (p = 0.048, One-way ANOVA with post LSD test; Fig. 3E). We also performed a Chi-Square Test for Trend to compare the RGC loss in NLRP3 knockout mice and controls (Supplementary Fig. S2), and the statistical results confirmed that more RGCs survived in NLRP3 knockout mice than in controls.

We determined the survival probability of RGCs using the Kaplan-Meier (K-M) estimator, a statistical measure commonly used in survival studies (see details in Methods). The K-M estimator modeled the time-courses of RGC survival in NLRP3^+/+^, NLRP3^+/−^, and NLRP3^−/−^ mice, and the results were compared by the log-rank test (Fig. 3F). We confirmed that RGC survival in NLRP3^−/−^ mice was better than in NLRP3^+/+^ (p < 0.001) and NLRP3^+/−^ mice (p = 0.001 in Log-rank test; Fig. 3F). Moreover, we showed that the median survival time of RGCs in NLRP3^+/+^and NLRP3^+/−^ mice were both 14 days, while the median time for NLRP3^−/−^ mice was 21 days (Fig. 3F). In other words, knocking out the *NLRP3* gene delayed RGC loss for one week.

### Axonal survival is also improved in NLRP3^−/−^ mice

**Figure 4:**
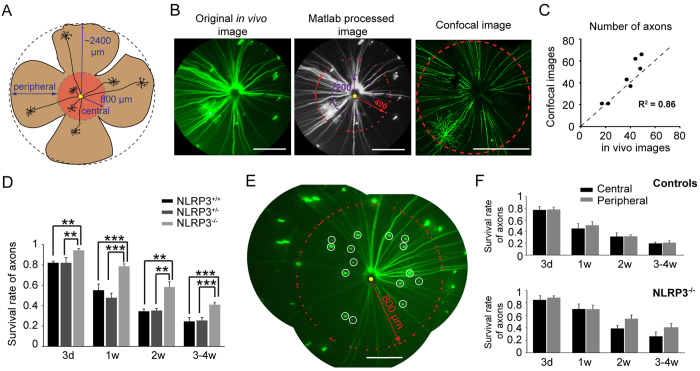
Axon loss was also delayed in the NLRP3^−/−^ mice following the pONC. (**A**) Diagram showing how we defined the central (red) and peripheral (brown) retina. (**B**) Axon counts by a customized Matlab program were validated by confocal imaging. The original image (left) was taken by the Micron III scope, processed in Matlab (middle), and then the mouse was sacrificed for confocal imaging (right). The number of axons was averaged from counts at the 200 (purple) and 400 μm (red) radii around the ONH (yellow circle). (**C**) The scatter plot shows that the numbers of axons counted from the Micron images and the confocal images were highly correlated. N = 7retinas, R^2^= 0.86. The unity line is shown as dashed. (**D**) The survival rate of axons in the NLRP3^−/−^ mice was much higher than control groups (NLRP3^+/−^ and NLRP3^+/+^). **p < 0.01, and ***p < 0.001 in one-way ANOVA with post LSD test. (**E**) Somas and their axons within 800 μm radius from the ONH were counted as the number of axons in the central retina (white circles). Axons in the peripheral retina were counted at 800 μm radius (marked in red by Matlab). Scale bars: 400 μm. (**F**) The survival rates of axons in the central area exhibited no significant difference compared to peripheral areas in both control mice (NLRP3^+/+^ and NLRP3^+/−^ mice, N = 8) and NLRP3^−/−^ mice (N = 6).

The RGC somas studied through the above analysis were mainly from images taken from what we termed the central retina, which we estimated covered about 40% of the retina as illustrated in Fig. 3A. In order to quantify RGC loss in more peripheral regions, we used axon counts (Fig. 4). Because axons converge on the ONH, we were able to analyze the axon numbers from the entire retina (Fig. 4A). First, we determined whether our axon counts from *in vivo* images provided an accurate measure of axon survival (Fig. 4B,C). *In vivo* images of the mouse retinas were taken before they were sacrificed for confocal imaging (n = 7 mice). We compared axon counts from the *in vivo* images and from confocal images of the same retinas (Fig. 4B) and found a strong positive correlation between the two methods (R^2^ = 0.86, n = 7 retinas, Fig. 4C).

We showed that the trend in axon loss was similar to the trend in RGC loss reported above (Fig. 4D). In the NLRP3^+/+^ mice, we counted a total of 149 axons from five retinas. At 3-d pONC, 82 ± 2% of axons survived (Fig. 4D), similar to the soma survival of the NLRP3^+/+^ mice shown in Fig. 3D (78 ± 3%, p = 0.3, Student’s *t*-test). The tendency for cell survival to be greater in NLRP3^−/−^ than in NLRP3^+/+^ and NLRP3^+/−^ mice was found for axons, just like for somas. In the NLRP3^−/−^ mice, we counted a total of 322 axons and 94 ± 2% of axons remained in NLRP3^−/−^ mice (n = 9) at 3-d after the pONC injury, much higher than in NLRP3^+/+^ (p = 0.004) and NLRP3^+/−^ mice (p = 0.006, one-way ANOVA with post LSD test; Fig. 4D). The axon survival rate was 79 ± 3% in NLRP3^−/−^ at 1-w (p < 0.001 compared to NLRP3^+/+^ and NLRP3^+/−^), and remained high at 2-w (58 ± 5%), compared to NLRP3^+/+^ (p = 0.002) and NLRP3^+/−^ mice (p = 0.003, One-way ANOVA with post LSD test; Fig. 4D). After four weeks post pONC, substantial axon loss was found in NLRP3^−/−^ mice (41 ± 2%), but remained lower than for the NLRP3^+/+^ and NLRP3^+/−^ mice (p < 0.001, One-way ANOVA with post LSD test; Fig. 4D). Both sets of data thus point to improved RGC survival following pONC injury in NLRP3^−/−^ mice.

To investigate whether axon loss was location-dependent, we compared the axon loss from the central and peripheral retina (Fig. 4A,E). Somas with their axons terminating within 800 μm of the ONH were counted as central, while axons that crossed the 800 μm radius counted as peripheral retina (Fig. 4E, note their somas may not be seen due to the limited field of view). Because heterozygous NLRP3^+/−^ mice showed no significant difference from NLRP3^+/+^ mice (p = 0.1, Fig. 4D), we combined them into one control group for Fig. 4F. For the control group, we found no difference in RGC loss between central and peripheral retina (n = 8 mice; p = 0.1, Two-way ANOVA; Fig. 4F). As early as 3-d post pONC, the survival rate of axons in the central area was 77 ± 6%, similar to the periphery area (78 ± 4%). The number of axons from both areas continued to decrease following a similar pattern (Fig. 4F). In NLRP3^−/−^ mice, the central and peripheral areas also exhibited similar protective effects (n = 6 mice, p > 0.05, Two-way ANOVA; Fig. 4F).

## Discussion

Retinal microglia activate in response to disturbances of the surrounding micro-environment such as pathogen-associated molecular patterns (PAMPs), damage-associated molecular patterns (DAMPs), bacterial pore-forming toxins and extracellular ATP^1^. In our study, the early activation of microglia was induced by optic nerve damage and spread retrogradely to the neural retina, consistent with previous findings (e.g.^11^,^12^). The danger signals released from injured axons activate the NLRP3 inflammasome to initiate and amplify the pro-inflammatory responses. For example, ER stress induced by diabetes and neurotoxicity in rodent retina activated NLRP3 inflammasome through thioredoin-interacting protein in Müller glial cells^21^,^23^. Zhong and his colleagues (2013) showed that extracellular ATP, considered a danger signal, activated the P2X7 receptor, triggering the activation of NLRP3 inflammasome in macrophages^24^. DAMPs could also activate the NLRP3 inflammasome and caspase-8 pathways following injury induced by temporary retinal ischemia^22^.

The NLRP3 inflammasome has been considered a key component in amplifying retinal inflammation^18^,^22^. We demonstrated that deletion of *NLRP3* reduced caspase-1 and IL-1β in the mouse retina (Fig. 2D–F). In fact the baseline level of caspase-1 and IL-1β was unchanged in NLRP3^−/−^ mice compared to controls (Fig. 2D–F). A recent study showed that inhibition of caspase-1 reduced IL-1β expression to baseline post retinal ischemia injury, while inhibition of caspase-8 further down-regulated the level of IL-1β, suggesting that IL levels could be regulated by both NLRP3-dependent and other pathways^22^.

Previous studies showed that NLRP3 inflammasome played a destructive role in dry AMD, with disease progression inhibited by blocking NLRP3 or downstream IL-18 and IL-1β^9^,^10^. Inhibition of caspase-1 also attenuated the damage from a short-term ischemic insult^22^. A destructive role for the NLRP3 inflammasome has also been reported for diabetic retinopathy, temporary ischemia, and NMDA neurotoxicity^21^,^22^,^23^. However, NLRP3 inflammasome could also down-regulate the synthesis of vascular endothelial growth factor through IL-18 to provide protection in a mouse model of wet AMD^7^,^8^. Here we have demonstrated that RGC survival was prolonged in NLRP3^−/−^ mice (Figs 3 and 4), supporting the idea that activation of NLRP3 inflammasome results in RGC loss following the pONC injury. We analyzed RGC survival using both soma counts and axon counts (Figs 3 and 4). The trend in axon loss corresponded with the trend in soma loss for both control and knockout mice, except that we found higher axonal survival compared to soma survival at 4-w post pONC (Figs 3 and 4). About 25% of axons survived in wildtype mice, a substantial decline certainly but a survival rate higher than for somas (13%, P = 0.05, Student’s *t*-test, Figs 3E and 4D). A similar discrepancy was observed for the NLRP3 knockout mice. This difference in long-term axon versus soma survival might result from a bias in the sample of somas visible through the Micron III system such as that somas of the peripheral retina were not tracked.

Some studies including ours suggested that RGC loss is type- and location-dependent following optic nerve injury or disease^25^,^26^,^27^,^28^. In this study we found no statistical difference between axon loss in the peripheral and central areas (Fig. 4F). In addition, the small error bars in Fig. 3D showed that individual retinas of each experimental group, regardless of type and number of RGCs labeled, exhibited similar RGC loss/survival following crush injury, arguing that activation of NLRP3 inflammasome may have affected RGC survival across the board. However, this does not rule out the possibility that, when studied at finer granularity, certain areas might be found to be more susceptible to the effects of pONC injury. It is also possible that the soma loss we observed reflects a bias towards specific (perhaps large soma) RGCs. High-resolution imaging of transgenic mice which have specific RGC types labeled would be needed to reveal more details in type- and location- dependent RGC loss brought about by optic nerve disease or injury.

In summary, our study supports the notion that the presence of NLRP3 accelerates cell loss in optic nerve injury, which establishes a foundation from which to examine the underlying neuroinflammatory mechanisms and a model system in which to test drugs targeting the NLRP3 inflammasome for preservation of vision in optic neuropathy.

## Methods

### Animals

C57BL/6 wild-type (WT), Thy-1-YFP transgenic mice (003782; The Jackson Laboratory, Bar Harbor, ME), and NLRP3^−/−^ mice (021302; The Jackson Laboratory) of either sex were used in this study. Thy-1-YFP H line, NLRP3^−/−^ mice were genotyped by polymerase chain reaction using forward primers to detect mutant: 5′-TGCCTGCTCTTTACTGAAGG-3′, and wide type: 5′-TCAGTTTCCTTGGCTACCAGA-3′; and the common reverse primer: 5′-TTCCATTACAGTCACTCCAGATGT-3′^29^. NLRP3^−/−^ mice (2–6-months old) were crossed with Thy-1-YFP mice, which had a small number of RGC labeled^30^,^31^, permit visualization of RGCs *in vivo*. All animal procedures conformed to the guidelines on the Use of Animals from the NIH and the Society for Neuroscience and were approved by the Northwestern University Institutional Animal Care and Use Committee.

### The Mouse Model of Partial Optic Nerve Crush (pONC) Injury

The partial optic nerve crush surgery was performed as described previously^25^,^32^,^33^. In brief, mice were anesthetized with an intraperitoneal injection of ketamine (100 mg/kg; Lloyd, Inc., Shenandoah, IA) and xylazine (10 mg/kg; Butler Schein Animal Health, Dublin, OH). A small incision was made in the superior and lateral conjunctiva and then a gentle dissection was made with fine forceps (Dumont #5B, World Precision Instruments, Sarasota, FL) to expose the optic nerve. The optic nerve was clamped with self-clamping forceps (#N7, World Precision Instruments) at a site about 1 mm behind the globe for 3 seconds (also see Fig. S1). Mice were kept on a heating pad (Sunbeam, Boca Raton, FL) until fully awake.

### *In vivo* Imaging

*In vivo* images of retinas were taken by a Micron III fundus scope (Phoenix Research Laboratories, Pleasanton, CA). Mice were anesthetized as described above and pupils dilated using 1% tropicamide (Akom, Lake Forest, IL) and 2.5% phenylephrine hydrochloride (Bausch & Lomb, Tampa, FL). After the mouse head was fixed in position by ear bars, 2.5% hypromellose ophthalmic demulcent solution (HUB Pharmaceuticals, Rancho Cucamonga, CA) was applied on the cornea. Retinal images were taken before pONC surgery, which served as the baseline, and then repeatedly taken at different time points post pONC.

The number of axons was counted automatically by a customized Matlab program (Mathworks, Natick, MA). The location of the center of the optic nerve head in the image was first selected, and the signal intensity measured radially at 200 and 400 μm around this point. Local maxima along these signal intensity profiles were classified as axons. The images were then examined by two observers to validate the automatic counts. Contrast and brightness of the images were adjusted in Photoshop (Adobe; San Jose, CA), and the numbers of axons were counted manually. The axon count was the average of the Matlab and manual counts (the difference between the two methods was typically less than 5%). To compare axonal loss between central and peripheral areas of the retina, the region within 800 μm of the optic nerve head (ONH) was defined as the central retina and the rest as the peripheral retina.

The survival rate of RGC somas and axons was calculated as:


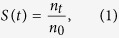


where S(t) represents the survival rate at time t, n_0_ is the number of RGC somas/axons at the time point before the pONC injury (i.e., baseline), and n_t_ is the number of RGCs at time t after the pONC crush.

### Modeling of RGC Survival

In order to estimate the probability of RGC survival, the Kaplan-Meier (K-M) estimator was applied (SPSS Inc., Chicago, IL)^34^,^35^.


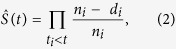


where 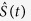
represents the K-M estimate at time *t*, *t*_*i*_ is the observed time point, *n*_*i*_ is number of RGCs at risk at time *t*_*i*_, *d*_*i*_ is the number of RGC deaths at *t*_*i*_. A log-rank test was applied to compare the survival distributions of different groups^36^.

### Retinal Immunohistochemistry

Immunostaining and Western Blots were performed as described previously^26^. Primary antibodies included anti-NLRP3 (1:1000 dilution, Abcam ab17267, Cambridge, MA), anti-Iba-1 (1: 1000, Abcam ab5076), anti-caspase-1 (1:250, Abcam 1872), anti-IL-1β (1:500, R&D Systems, Inc., MN), anti-ASC (1:100, Millipore 04-147), and anti-GAPDH (1:1500, Millipore MAB374). Alexa Fluor-conjugated secondary antibodies were used (1:1000, Invitrogen, Carlsbad, CA), and slides were coverslipped with Vectashield antifade mounting medium (Vector Laboratories, Burlingame, CA), with some containing 4′, 6-diamidino-2-phenylindole (DAPI, Vector Laboratories). Images were captured using a Zeiss LSM5 Pascal confocal microscope (Zeiss, Thornwood, NY). In each flat-mounted retina, we counted microglial cells that co-localized Iba-1 and NLRP3 in eight 10^4 ^μm^2^ areas and expressed them as the percentage of Iba-1 + NLRP3 cells to total Iba-1 + microglia cells.

All data are presented as mean ± SEM. Student’s *t*-test was applied to examine the differences for paired groups, One-way ANOVA with LSD *post hoc* test to compare means of three or more samples, and Two-way ANOVA to examine two different categorical independent variables. The coefficient of determination, R^2^, was used to examine the correlation between the results from *in vivo* imaging and confocal imaging methods.

## Additional Information

**How to cite this article**: Puyang, Z. *et al.* Retinal Ganglion Cell Loss is Delayed Following Optic Nerve Crush in NLRP3 Knockout Mice. *Sci. Rep.* **6**, 20998; doi: 10.1038/srep20998 (2016).

## Supplementary Material

Supplementary Information
