# Supplementary material for: Retinal Ganglion Cell Loss is Delayed Following Optic Nerve Crush in NLRP3 Knockout Mice
Source: Sci Rep. 2016 Feb 19;6:20998. doi: 10.1038/srep20998 (PMC4759563; doi:10.1038/srep20998)
Supplement: Supplementary Information [file srep20998-s1.pdf]

Supplementary Information

Retinal Ganglion Cell Loss is Delayed Following Optic Nerve Crush in NLRP3 Knockout Mice

Zhen Puyang[1, 2, 3,+], Liang Feng[3, 4,+], Hui Chen[3], Peiji Liang[1], John B. Troy[2], and Xiaorong Liu[3, 4,*]

Figure S1, Related to Figure 1. The mouse model of partial optic nerve crush (pONC). (A) Schematic diagram of the optic nerve crush surgery. Different laboratories adopted slightly different techniques to induce acute RGC and axon loss. For example, Libby and his colleagues (2005) clamped the optic nerve approximately 0.5 mm from the globe with self-closing jeweler's forceps for 4 s [1]. Templeton and Geisert (2012) used Dumont #N7 cross-action forceps (#RS-5027; Roboz) to grasp the optic nerve approximately 1–3 mm from the globe for 10 s [2]. Caution was made not to cut into the underlying musculature or the supplying vasculature [1,2]. (B) Representative images of the cross-sections of the optic nerve head (ONH). After pONC, significant axon loss was observed with disruption of the myelin sheath (curved arrow), hyper-dense axoplasm (arrow), vacuolization (arrowhead) and glial scar (asterisk). (C) The total number of axons in the optic nerve was counted at before (control), and 1-day and 1-week post pONC. Control: n = 9 mice; 1 day: n = 5; 1 week: n = 5; ***$p < 0.001$ in One-way ANOVA post-LSD test. Data are represented as mean ± SEM. Note the axon loss following the pONC may vary among different studies, depending on the force and duration of the crush injury [1-4].

(D - E) Retinal cross-sections were immuno-labeled with antibodies against Brn-3a and Brn-3b, RGC markers. The numbers of Brn-3b (D) and Brn-3a (E) positive cells decreased after pONC, confirming the RGC loss with time. (F - H) The inner retina exhibited largely normal structure following the pONC. Retinal cross-sections were immuno-labeled with antibodies against calretinin (F) and calbindin (G), markers for amacrine and ganglion cells, and tyrosine hydroxylase (TH), a marker for dopaminergic amacrine cells (H).

Figure S2, Related to Figure 3E. A different statistical analysis showed that more RGCs survived in NLRP3$^{-/-}$ mice than in controls (NLRP3$^{+/-}$ and NLRP3$^{+/+}$). The total number of RGCs for each experimental group is indicated above the baseline (100% survival) data. The percentage of surviving RGCs from each experimental group was analyzed at different time points post pONC injury. A Chi-Square Test for Trend was performed to compare the difference of two categorical samples and the results were consistent with Figure 3E.

References

1. Libby, R.T., Li, Y., Savinova, O.V., Barter, J., Smith, R.S., Nickells, R.W., and John, S.W. (2005). Susceptibility to neurodegeneration in a glaucoma is modified by Bax gene dosage. PLoS Genet *1*, 17-26.

2. Templeton, J.P., and Geisert, E.E. (2012). A practical approach to optic nerve crush in the mouse. Mol Vis *18*, 2147-2152.

3. Leung, C.K., Weinreb, R.N., Li, Z.W., Liu, S., Lindsey, J.D., Choi, N., Liu, L., Cheung, C.Y., Ye, C., Qiu, K., et al. (2011). Long-term in vivo imaging and measurement of dendritic shrinkage of retinal ganglion cells. Investigative ophthalmology & visual science *52*, 1539-1547.

4. Sharma, T.P., McDowell, C.M., Liu, Y., Wagner, A.H., Thole, D., Faga, B.P., Wordinger, R.J., Braun, T.A., and Clark, A.F. (2014). Optic nerve crush induces spatial and temporal gene expression patterns in retina and optic nerve of BALB/cJ mice. Mol Neurodegener *9*, 14.

Supplementary Fig. S1

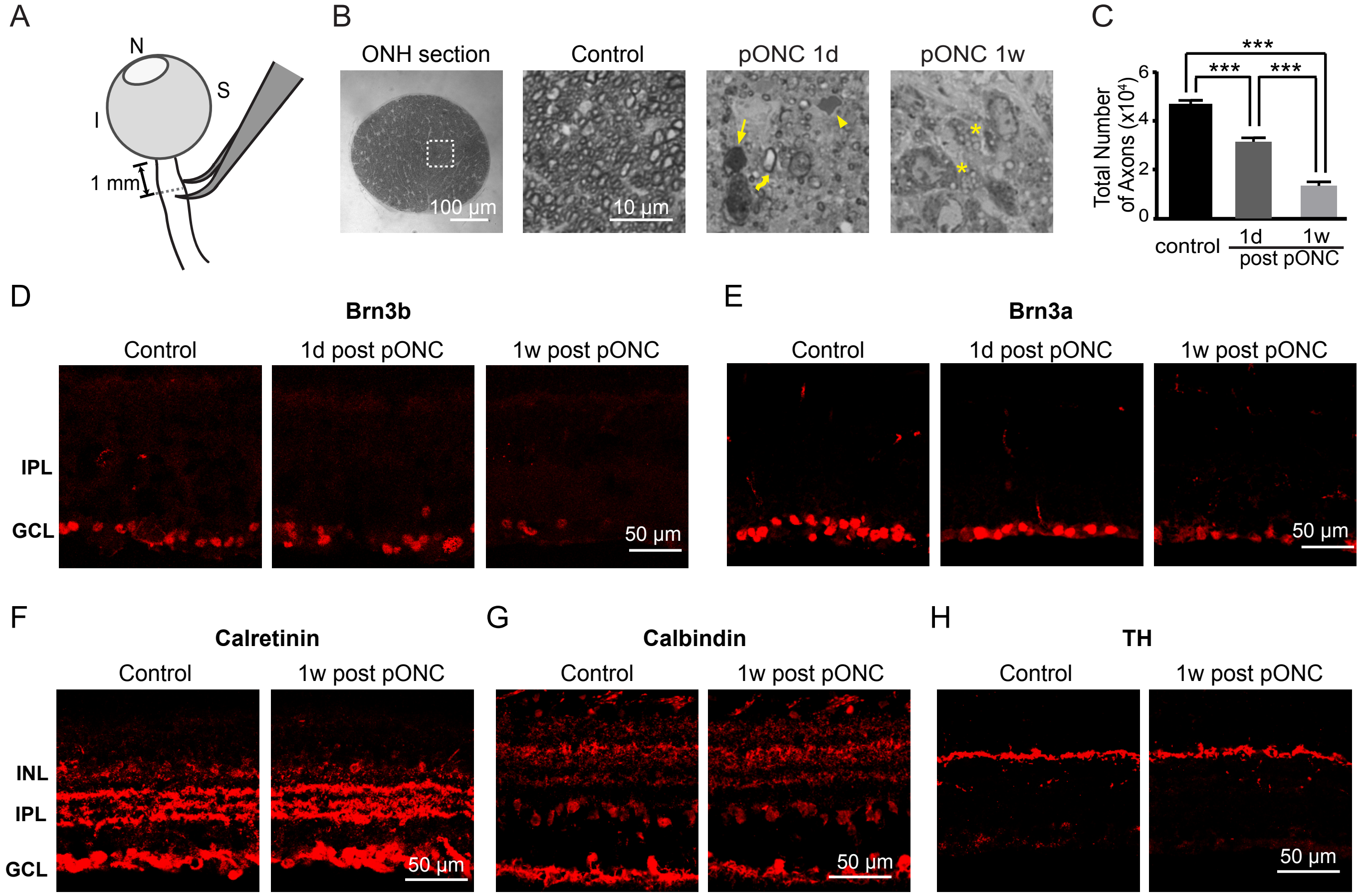

Supplementary Fig. S2

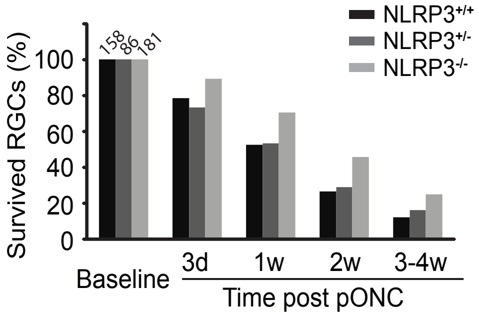

Chi-Square Test for Trend

$NLRP3^{-/-}$ vs. $NLRP3^{+/+}$: P = 0.0008

$NLRP3^{-/-}$ vs. $NLRP3^{+/-}$: P = 0.046

$NLRP3^{+/+}$ vs. $NLRP3^{+/-}$: P = 0.48
